# Integration of a high-resolution melt curve assay into a commercial quantification kit for preliminary identification of biological mixtures

**DOI:** 10.1007/s00414-025-03427-z

**Published:** 2025-02-01

**Authors:** Chastyn Smith, Andrea L. Williams, Hannah E. Wines, Darianne C. Cloudy, Jordan O. Cox, Sarah J. Seashols-Williams, Edward L. Boone, Tracey Dawson Green

**Affiliations:** 1 Department of Forensic Science, Virginia Commonwealth University, 1015 Floyd Avenue, Richmond, VA 23284 USA; 2 Integrative Life Sciences, Virginia Commonwealth University, 1000 Cary Street, Richmond, VA 23284 USA; 3 Department of Statistical Sciences & Operations Research, Virginia Commonwealth University, 1015 Floyd Avenue, Richmond, VA 23284 USA

**Keywords:** Forensic science, Mixture screening, High-resolution melt curve (HRM), Support vector machine modeling (SVM), Linear discriminant analysis, Investigator Quantiplex^®^ kit

## Abstract

In recent years, DNA analysis techniques have drastically increased in sensitivity, allowing for low template DNA samples to be more easily detected and used for identification. Since the problems inherent in low template DNA are exacerbated in DNA mixture samples, it would be advantageous to incorporate an assay earlier in the DNA workflow that could detect a mixture and, potentially, determine the number of contributors. Some real-time PCR instruments have high-resolution melt curve analysis (HRM) capabilities, allowing for an opportunity to integrate an HRM screening assay into a commercial DNA quantification kit. This work describes the integration of a mixture screening HRM assay using STR loci D5S818 and D18S51 into Qiagen’s Investigator Quantiplex^®^ kit. The integrated Quantiplex^®^-HRM assay was tested on two qPCR platforms: The Rotor-Gene^®^ Q and the QuantStudio™ 6 Flex. Data from this assay was analyzed using linear discriminant and support vector machine analyses for sample classification. When HRM curve data from the Rotor-Gene^®^ Q was used for classification, the integrated assay exhibited an overall accuracy of 89.39%, correctly classifying 87.5% of single source samples and 100% of mixtures. When HRM curve data from the QuantStudio™ 6 Flex was used for classification, the integrated assay exhibited an overall accuracy of 87.88%, correctly classifying 87.5% of single source samples and 90% of mixtures. The overall accuracy of the integrated Quantiplex^®^-HRM assay on both instruments met our goal of ≥ 80% accuracy, demonstrating the viability of the assay to detect mixtures when integrated into a commercial quantification kit.

## Introduction

The forensic DNA field seems perpetually faced with the challenge of labor intensive and time-consuming casework alongside the criminal justice community’s demand for more timely analysis of evidence from crime scenes. Over time, forensic analysis methods for DNA amplification and STR profiling have greatly increased in sensitivity, allowing for samples containing degraded or trace amounts of DNA, such as touch DNA, to be analyzed. Touch DNA samples are those that result from the transferal of minute amounts of biological material from the body when contact is made with a surface [1, 2]. These touch DNA samples often contain low levels of template DNA (less than 100pg available for STR amplification), which becomes problematic during PCR as some target regions may be preferentially amplified over others [3]. This can result in STR allelic drop in, drop out, or peak imbalances [4] in the resulting profiles, making their interpretation more difficult. This process is further complicated because these touch DNA samples are inherently prone to mixtures – i.e. samples that include DNA from more than one contributor – since they originate from surfaces that may have been touched by numerous individuals [5].

Unfortunately, in the current forensic DNA workflow, the number of contributors in a sample is not revealed until the final step of analysis – a process that can easily take weeks from start (initial sample evaluation and serology) to finish (case report issued). When a mixture is present, along with low amounts of DNA, resulting data often includes one or more of the contributors’ allele peaks falling below the analytical threshold often leading to “inconclusive” reporting. Additionally, because this information is not available until endpoint analysis, it is not possible to make earlier analytical adjustments to the protocols or workflow that may serve to increase the likelihood of generating a profile with a distinguishable minor contributor. While reamplification of a low, mixed DNA sample may be possible, it is time consuming. Additionally, with low template or touch samples, the samples are more often consumed during initial testing leaving little or no remaining DNA for a second analysis. Due to these potential complications, many laboratories elect not to routinely accept, process, or interpret touch DNA samples [6]. A screening assay that could detect the contributor nature of a sample and potentially provide early exclusionary information earlier in the forensic DNA workflow would give analysts more options for processing low-level touch DNA samples, which may, in turn make laboratories to be less reluctant to process them. Knowledge of the presence of a mixture (versus a single source sample) could redirect the DNA workflow in an effort to improve the number of passing profiles obtained during the first round of testing, subsequently reducing retest rates.

The ability to distinguish between single source and mixed biological samples may be most easily introduced at the real time PCR (qPCR) stage. Real time PCR instruments combine the functions of a thermal cycler and fluorometer; they are routinely used by forensic DNA laboratories to detect the amount of amplifiable human DNA in a sample, using commercialized qPCR kits. Regardless of the exact model used, their multi-channel fluorescent detection, coupled with amplicon melt capabilities, allows for an opportunity to integrate additional assays into existing quantification kits [7–10] – thereby providing more information about evidentiary samples, earlier in the workflow. The Applied Biosystems™ 7500 (ABI 7500, Thermo Fisher, Waltham, MA) is the most frequently used qPCR instrument in the forensic community historically due to its broad acceptance of producing reliable and accurate data [8]. It is a five-color platform that allows for customizable melt curve protocols to be performed and analyzed with its melt curve analysis (MCA) software [11, 12]. The Rotor-Gene^®^ Q (QIAGEN, Hilden, Germany) is another instrument that offers quantification, amplification, and high-resolution melt (HRM) curve analysis, and it is equipped with seven dye channels; one being the specially tuned extended green HRM channel. This, combined with its comprehensive HRM software, allows for a relatively simple melt curve analysis [13, 14]. The QuantStudio™ 5 system and 6 Flex system (Thermo Fisher) are qPCR platforms that have a decoupled six color and coupled five color filter set, respectively, that allows for the addition of custom dyes and post-PCR HRM [15]. Further, these QuantStudio™ models offer on-board melt curve analysis with its custom HRM software module [15, 16]. The QuantStudio™ models are newer than the Rotor-Gene^®^ Q and are supported for use with several common forensic DNA quantification kits [17–21].

The availability of melt curve (or dissociation) analysis with most modern qPCR instruments affords a functionality that can be easily exploited to gain more information about a sample at this early stage. The melt curve function detects and measures the change in fluorescence of the amplified products over time as the temperature is slowly increased. Thus, as the temperature increases, the DNA dissociates (melts), corresponding to differences in DNA composition such as length and sequence, suggesting it may be possible to use these curves to differentiate between various alleles or genotypes at a given genetic locus and even distinguish mixtures [22–27]. For example, Kuehnert et al. determined that the intercalating dye EvaGreen^®^ (Biotium, Freemont, CA) detected in the green channel at 510 nm, could be used to detect and distinguish single source STR amplicons from mixed contributor amplicons using the HRM channel of the Rotor-Gene^®^ Q [23].

In fact, several previous studies have utilized STR loci D5S818 and D18S51 for development of HRM-based mixture detection assays [23, 26, 28, 29]. D5S818 has small amplicon sizes (115–178 bp) and a small range of repeats (6–18) whereas, the STR locus D18S51 has larger amplicons (262–342 bp) and a larger range of repeats (7–27) [26, 30]. The difference in amplicon length prevents overlap of the resulting D5S818 and D18S51 melt curves, allowing these loci to be easily duplexed (amplified and melted simultaneously in a single reaction). In conjunction with machine learning algorithms, such as linear discriminant analysis (LDA) and Support Vector Machines (SVM), this approach can be a very flexible technique for making sample contributor predictions. However, for practical application, an assay of this type would be best if incorporated into the existing qPCR (quantification) step, rather than as an additional, stand-alone process.

The Investigator Quantiplex^®^ quantification kit (Quantiplex^®^, QIAGEN, Hilden, Germany) is a commercially available qPCR kit that quantifies human genomic DNA in a sample. Quantiplex^®^ has only one target – a human target in the FAM dye channel - and an internal PCR control in the VIC dye channel. The goal of the work described herein was to establish foundational knowledge for the direct integration of a melt curve assay into a qPCR kit, the Quantiplex^®^ quantification kit, as a way to provide additional data that could be used to assign evidentiary DNA samples to either a single source genotype or identify it as a mixture (containing DNA from multiple contributors). With the success of this work and the fundamental principles for an integrated qPCR-HRM assay outlined, this work has been used as a premise for more complex and informative integrated qPCR-HRM assays, specifically the integrated Quantifiler Trio™-HRM assay.

## Methods

### Sample collection & STR profile generation

Buccal swab DNA extracts were utilized for this study and were collected according to a university-approved Institutional Review Board (IRB) protocol (HM20002931). Nearly 400 buccal samples were collected in order to identify 10–20 samples that share five-to-seven individual common genotypes for either the D5S818 STR locus or the D18S51 STR locus. Sample DNA was extracted and quantified following the protocol described by Cloudy et al. [29]. Sample DNA extracts ranged in concentration from 0.6ng/µl − 23ng/µl. Sample reference profiles were then developed by amplifying and separating and detecting STR products following the protocol referenced in Cloudy et al. [29]. Samples were sorted into known reference genotype groups based on the resulting D5S818 and D18S51 genotypes. Samples that represented the seven most common D5S818 and D18S51 genotypes in the sampled population were used for testing in the studies detailed below. This included the genotypes (10,11), (11,11), (11,12), (11,13), (12,12), (12,13), and (13,13) for D5S818 and (12,13), (12,14), (12,15), (12,16), (13,14), (13,16), and (14,15) for D18S51.

### Integration and functional testing of the Quantiplex®-HRM assay

To evaluate the success of the melt curve assay within the qPCR-based quantification step of the forensic DNA workflow, the D5S818 and D18S51 primers and EvaGreen^®^ dye were integrated into the Quantiplex^®^ kit and tested on the Rotor-Gene^®^ Q and QuantStudio™ 6 Flex qPCR platforms using HRM analysis. Integrated Quantiplex^®^-HRM reactions included a 16.16 µl master mix comprised of 7.36 µl of the Quantiplex^®^ primer mix, 7.36 µl of the Quantiplex^®^ reaction mix, 0.16 µl of 100µM forward and reverse primer for each STR locus [29], and 0.8 µl 20x EvaGreen^®^ intercalating dye. To each reaction, 1 µl of template DNA, directly from the unquantified extract, was added for a total reaction volume of 17.16 µl. In order to assure proper amplification of the Quantiplex^®^ targets, the thermal cycling program was slightly altered. Thermal cycling parameters used for the integrated Quantiplex^®^-HRM assay included a 10 min 95 °C denaturation followed by 40 cycles of 95 °C for 5s and 60 °C for 30s. Following amplification, samples underwent a transition cycle consisting of 72 °C for 2 min, 95 °C for 20s, 55 °C for 20s and 56 °C for 2 min, after which the amplicons were melted. Amplicon melt parameters for the Rotor-Gene^®^ Q were identical to those referenced in Cloudy et al. [29]. The resulting melt curves showed D5S818 and D18S51 amplicons melting from 60 °C to 78.49 °C and 78.5 °C to 95 °C, respectively. The QuantStudio™ 6 Flex melt program included a ramp from 60 °C to 95 °C using the continuous setting with a ramp rate of 0.015 °C/s, which allowed for maximum resolution. The resulting melt curves showed D5S818 and D18S51 amplicons melting from 60 °C to 77.481 °C and 77.501 °C to 84.00 °C, respectively.

In order to determine if the Quantiplex^®^ amplicons themselves produce melt products when the transition and melt cycles were added to the amplification parameters an additional set of Quantiplex^®^ standards were amplified on the Rotor-Gene^®^ Q using the manufacturer’s recommended reaction conditions (without STR primers or EvaGreen^®^ dye) and amplification parameters, but with the added transition and melt program described above. Resulting melt curves were qualitatively compared to those obtained when using the integrated Quantiplex^®^-HRM assay, as described above. To compare melt curves, 10 samples with D5S818 and D18S51 genotypes within the selected study set were amplified using the integrated Quantiplex^®^-HRM assay, as described above. The mean and standard deviation of the D5S818 and D18S51 primary melt peak temperatures were calculated and compared to those obtained in the initial evaluation studies described above using a two-tailed student’s t-test (α = 0.05).

In order to determine if alterations in Quantiplex^®^ reaction chemistry would affect resulting human DNA quantification estimates expected two sets of Quantiplex^®^ standard samples were analyzed across two separate Rotor-Gene^®^ Q runs using both the traditional Quantiplex^®^ chemistry (with half reactions) and the integrated Quantiplex^®^-HRM assay (described above). On each run, one set of standards were used to generate the standard curve while the other set of standards were evaluated as unknowns. Resulting standard curve quality metrics and inter-run variation were compared. The inter-run variation was determined by calculating the average differences in quantification values between runs and the percent differences observed in quantification values obtained for each standard sample from each run, as described above. The percent difference was calculated by dividing the absolute value of the difference in quantification values obtained across runs by the average.

### Single source vs. mixture prediction using the integrated Quantiplex®-HRM assay

Available DNA samples were split into training and validation sample sets and subsequently tested using the newly optimized integrated Quantiplex^®^-HRM assay and two qPCR platforms (Rotor-Gene^®^ Q the QuantStudio™ 6 Flex). The training set consisted of 101 single source DNA samples with D5S818 and D18S51 genotypes of interest (see above). Additionally, 10 1:1 two-person mixtures (made of contributors with genotypes of interest) were included in the training set. The validation set consisted of 56 single source samples, each having genotypes of interest at both loci, as well as 10 different 1:1 two-person mixtures. Negative derivative data from every temperature point along the entire melt curve for each tested sample was imported into the R statistical software and tested using prediction models. Three machine learning tools were tested within R statistical software to determine which model provided the highest genotyping classification rates for each qPCR platform used. These models were: Linear Discriminant Analysis (LDA), support vector machine with linear basis functions (SVM linear), and SVM with radial basis functions (SVM radial). Each algorithm used select data features to prevent overfitting. Confusion matrices were generated and subsequently used to determine the accuracy of the predictions. Single source typing prediction accuracies were determined for each locus by calculating the total number of samples classified as a single source genotype (Table 1, seen along the diagonal, regardless of whether the correct genotype was obtained) divided by the total number of single source samples tested in the validation set. Similarly, mixture accuracy was determined by dividing the number of mixture samples correctly classified by the total number of mixtures tested in the validation set. For combined accuracy of the integrated Quantiplex^®^-HRM assay, predictions for both STR loci tested were considered. If either STR locus was classified as a mixture for a given sample, then the final classification for that sample was indicated as a mixture. Finally, the number of samples that classified accurately (as either a single source or mixture) was divided by the total number of samples tested in order to determine the overall accuracy of the integrated Quantiplex^®^-HRM assay for both qPCR platforms.

**Table 1:** D5S818 confusion matrix example from prediction modeling algorithms

SVM Radial		Predicted Genotype							
		1011	1111	1112	1113	1212	1213	1313	Mix
Actual Genotype	1011	**0**	1	2	2	0	0	1	2
	1111	0	**3**	1	0	1	0	3	0
	1112	2	1	**1**	4	1	0	2	1
	1113	0	0	0	**1**	1	2	1	2
	1212	1	1	3	1	**0**	0	1	1
	1213	1	0	2	1	3	**0**	2	0
	1313	0	0	0	0	0	1	**3**	0
	Mix	0	0	0	1	0	0	1	**8**

*Sample numbers in bold, along the diagonal are those that were accurately predicted

## Results and discussion

### Integration and functional testing of the Quantiplex®-HRM assay

**Fig. 1:**
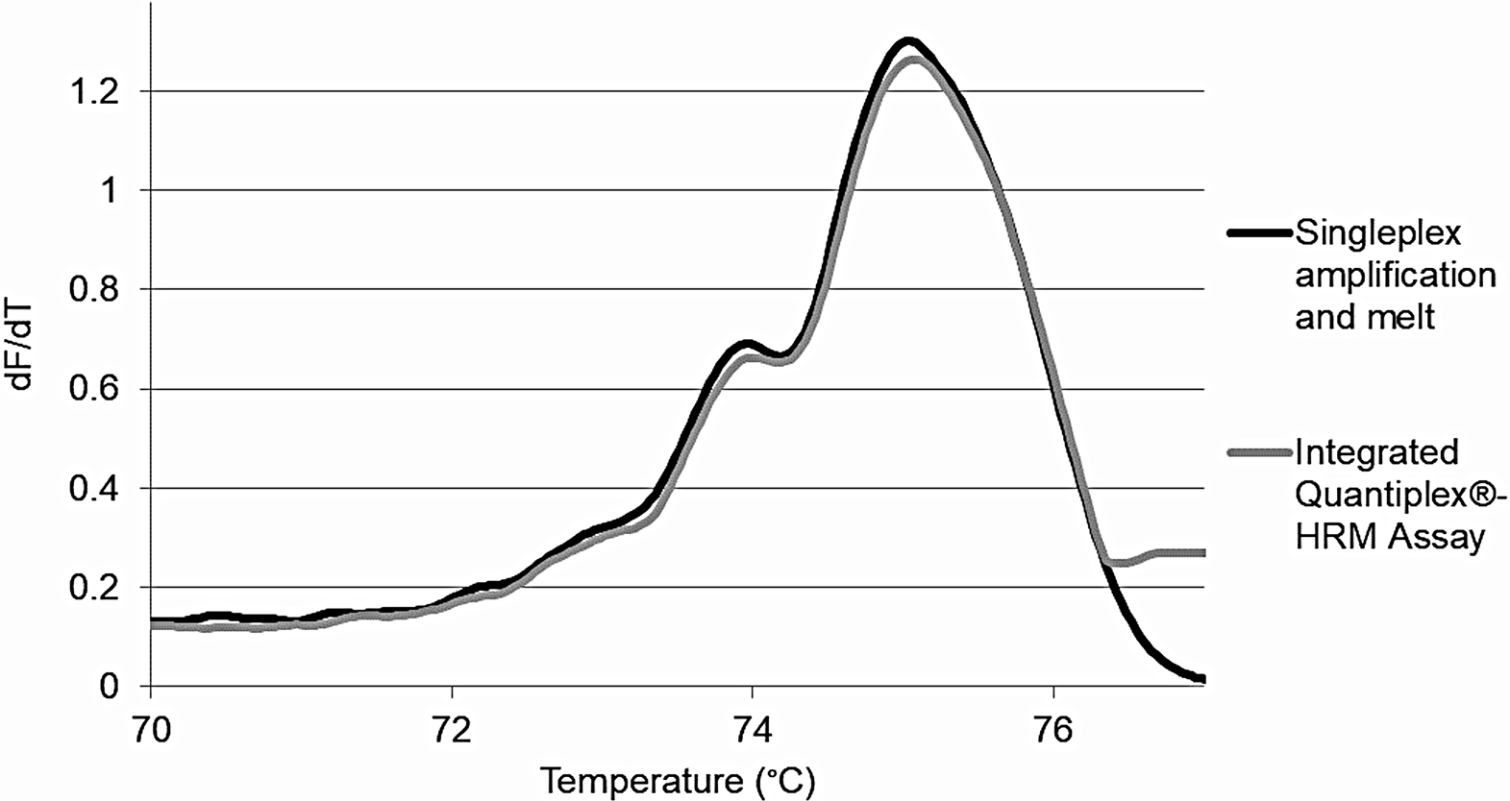
D5S818 melt curve for a single source sample using two different amplification/melt parameters on the Rotor-Gene Q^®^. dF/dT represents change in fluorescence level (positive or negative) with respect to per unit change (increase) in temperature. The integrated Quantiplex^®^-HRM assay melt of D5S818 produced a melt curve that is similar in fluorescence and overall curve morphology to that obtained using the singleplex amplification and melt

To evaluate the success of the melt curve assay within the qPCR-based quantification step of the forensic DNA workflow, the D5S818 and D18S51 primers and EvaGreen^®^ dye were integrated into the Quantiplex^®^ kit and evaluated on the Rotor-Gene^®^ Q. Initially, standard samples were tested on the Rotor-Gene^®^ Q platform and melt curves qualitatively evaluated to ensure that alterations to the reaction and amplification conditions did not affect the subsequent melt curves produced. Melt curves obtained were compared to those produced from initial evaluation studies. Samples amplified using the integrated Quantiplex^®^-HRM assay produced curves, in the correct STR amplicon melt range, that were indistinguishable from those developed previously using Cloudy et al. developed singleplex amplification and melt of the D5S818 and D18S51 amplicons (Fig. 1) [29]. Further, there were no significant differences in primary peak melt temperature when the samples amplified using the integrated assay were compared to those obtained using the singleplex amplification and melt of D5S818 and D18S51 (*p* = 0.8496 and 0.1895 for D5S818 and D18S51, respectively, data not shown). Similar observations were noted when melt curves were examined using the same testing conditions on the QuantStudio™ 6 Flex qPCR platform. Together, these data demonstrate that the Quantiplex^®^ chemistry does not alter the melt curves produced or contribute any additional melt products to the integrated assay.

**Fig. 2:**
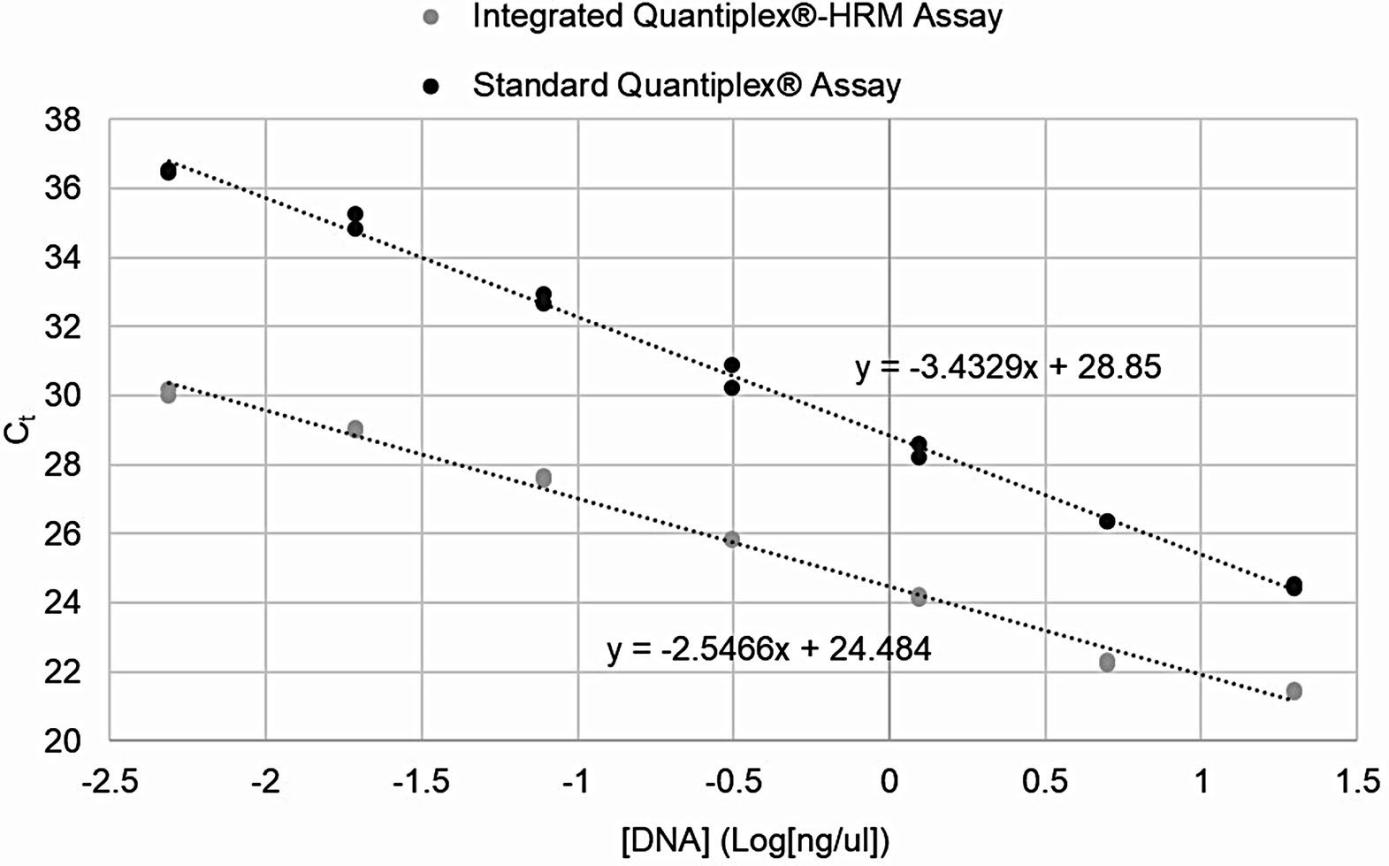
Representative integrated Quantiplex^®^-HRM assay standard curve and associated QC measures compared to the standard Quantiplex^®^ assay using the Rotor-Gene^®^ Q. Slopes obtained using the altered chemistry and reaction conditions were slightly higher than the expected range of the standard assay (-3.0 to -3.6). However, the Ct difference was consistent across all standards in the curve, thus quantification is not expected to be impacted

In addition to studying the effects of the altered Quantiplex^®^ reaction on the melt curves themselves, it was important to determine if the altered reaction conditions would impede the accuracy of the quantification reaction. When tested on the Rotor-Gene^®^ Q platform, R^2^ values and Y intercepts were unaffected by these alterations and consistently fell within the manufacturer’s expected values (data not shown). However, the slope was slightly higher (-2.55) than the expected range (-3.0 to -3.6) (Fig. 2). While this was unexpected, it was not expected to be problematic as the approximate 2 cycle change to the standard sample Ct values is observed consistently across all standards in the curve and thus, isn’t expected to alter resulting quantification values. In order to determine if this was true, quantification values from standards tested using the integrated Quantiplex^®^-HRM assay were compared to those obtained when the same standards were tested using the standard Quantiplex^®^ chemistry and reaction (Table 2). The values obtained when using the integrated Quantiplex^®^-HRM assay were not significantly different from those obtained using the standard Quantiplex^®^ chemistry and reaction (*p =* 0.2148). Further, these values were more similar to the expected values than were those obtained from duplicates run across multiple plates using the standard Quantiplex^®^ chemistry and reaction (inter-run variation). Likewise, the quantification values of experimental reference samples obtained from the integrated Quantiplex^®^-HRM assay were not significantly different from those obtained using the standard Quantiplex^®^ chemistry and reaction (*p* = 0.7685, data not shown). Similar observations were noted when melt curves were examined using the same testing conditions on the QuantStudio™ 6 Flex qPCR platform. Taken together, these data indicated that human DNA quantification accuracies are not impacted by the changes to the reaction and thermal cycling parameters.

**Table 2:** Quantiplex^®^ hDNA quantification vs. integrated Quantiplex^®^-HRM hDNA quantification

	Quantiplex^®^ reaction and amplification*	Quantiplex^®^ reaction with STR primers & amplification with transition & melt^♢^
Average difference between runs	0.5371 ng/µl	0.7808 ng/µl
Variation between runs	20.72%	17.74%

*based on multiple runs in Dawson-Green laboratory

^♢^as compared to standard Quantiplex^®^ and amplification run

*n* = 16

*p* = 0.2148

### Single source vs. mixture prediction using the integrated Quantiplex®-HRM assay

The new integrated Quantiplex^®^-HRM assay was tested to determine its ability to accurately distinguish single source from mixture samples (containing DNA from two contributors). The best prediction models for each STR locus tested using the Rotor-Gene^®^ Q were SVM radial and SVM linear for D5S818 and D18S51, respectively. For the QuantStudio™ 6 Flex SVM linear and SVM radial performed best for the D5S818 data and D18S51 data, respectively. Thus, all further calculations of prediction accuracy used the confusion matrices from these algorithms. Using only the D5S818 data, 94.46% and 89.29% of single source validation samples and 100% and 80.0% of mixtures were accurately predicted as such using the Rotor-Gene^®^ Q and QuantStudio™ 6 Flex, respectively. Alternately, using the D18S51 HRM data alone, 92.86% and 94.64% of single source samples and 100.0% and 60.0% of mixtures were accurately predicted as such using the Rotor-Gene^®^ Q and QuantStudio™ 6 Flex, respectively (data not shown). However, as the HRM assay incorporates two loci as a way to increase the power of discrimination in its predictions, a combined accuracy metric that overall classifies a sample as “mix” if one “mix” classification is obtained in either locus was used for overall prediction accuracies. With this approach, if a sample were to be inaccurately classified as single source at one locus and a mixture at the second locus, assuming the sample is single source may lead to combining of sample extracts and creation of artificial mixtures; thus, this interpretation is a more conservative approach. With both loci considered, 87.5% of single source samples and 100% of mixtures were correctly classified as such when using the integrated Quantiplex^®^-HRM assay on the Rotor-Gene^®^ Q, producing an overall accuracy of 89.39% (Table 3) in which 59 of the 66 samples tested were correctly classified. When the assay was tested in combination with the QuantStudio™ 6 Flex qPCR platform, 87.5% of the single source samples and 90% of the mixtures were correctly classified as such when using the same assay, producing an overall accuracy of 87.88% (Table 3) in which 58 of the 66 samples tested were correctly classified.

**Table 3:** Single source and mixture sample prediction accuracy using the integrated Quantiplex^®^-HRM assay

	Rotor-Gene^®^ Q	QuantStudio™ 6
Single source accuracy *n* = 56	87.50%	87.50%
Mixture accuracy *n* = 10	100.0%	90.00%
Overall accuracy	89.39%	87.88%

## Conclusion

The initial goal of this study was to evaluate an STR-based melt curve assay that could be incorporated into the qPCR quantification step in the forensic DNA workflow and used for characterizing human DNA evidence samples as either single source or a mixture of multiple contributors. Previous assay development began on the most commonly used qPCR platform in the forensic field, the ABI 7500, but further progression determined that the resolution capabilities of this platform were not suitable for STR-based melt analysis [31]. Thus, this paper furthered testing and evaluation of the Quantiplex^®^-HRM integrated assay on two qPCR platforms with high-resolution melt capabilities, the Rotor-Gene^®^ Q and the newest in the ABI qPCR series to be validated and marketed to the forensic DNA community, the QuantStudio™ 6 Flex. Testing of the Quantiplex^®^-HRM integrated assay, which included added STR primers, EvaGreen^®^ dye, and a transition cycle and melt that occur after the quantification, showed that resulting melt curves were reproducible and unaltered by the integration. Although slopes produced from the quantification standard curves using the Quantiplex^®^-HRM integrated assay were slightly higher than the expected range, the R^2^ values were within the acceptable quality ranges. Further, there were no significant or practical changes in the human DNA concentration values when those obtained using the new assay were compared to the values obtained when using the standard Quantiplex^®^ reaction. Lastly, and most importantly, the integrated Quantiplex^®^-HRM assay is able to accurately predict samples as either single source or mixture nearly 90% of the time, regardless of what qPCR platform was used. Thus, this assay provides a practical solution for identification of evidentiary mixture samples at the stage of quantification, without additional use of DNA sample and without additional time.

Overall, this work has successfully produced a qPCR-based melt curve assay for the prescreening identification of mixtures, and the assay has been demonstrated to be viable when integrated into a commercial quantification assay. Further, this introductory work has been used as a precursor to incorporate the developed melt curve assay into a commercial quantification kit that is more commonly used in the forensic DNA community, the Quantifiler™ Trio kit [32]. Moving forward, testing additional machine learning models may help identify a method that will improve genotyping classification for single source DNA samples. With that, future work should include additional developmental validation, such as testing mixtures with multiple contributors and different ratios, and expansion of the training set (to include all common genotypes at both STR loci and additional mixtures). Ultimately, implementation of this assay into a forensic DNA laboratory alongside a user-friendly reporting interface will provide forensic analysts with more information about the nature of evidentiary samples, earlier in the workflow, without the need for any additional steps in the workflow. This, in turn, could save valuable examiner time as well as reduce consumable expenses for the laboratory.

## Data Availability

The datasets generated during and/or analyzed during the current study are available from the corresponding author on reasonable request.
